# Comparative Evaluation of Push‐Out Bond Strength Between CAD/CAM‐Milled and Anatomically Customized Fiber Posts: An In Vitro Study

**DOI:** 10.1155/ijod/4637841

**Published:** 2026-09-17

**Authors:** Camila Orlinda Andrade Gusmão, Vanessa Inácio Celestrino, Franciele Floriani, Amirali Zandinejad, Carlos Alberto Jurado, Washignton Steagall, Rubens Nisie Tango, Alfredo Mikail Melo Mesquita

**Affiliations:** ^1^ Master in Prosthodontics, Universidade Paulista (UNIP), São Paulo, Brazil, unip.br; ^2^ Department of Prosthodontics, University of IOWA, Iowa City, Iowa, USA, uiowa.edu; ^3^ Department of Prosthodontics, School of Medicine and Dentistry, University of Rochester, New York, USA, rochester.edu; ^4^ School of Dental Medicine, Ponce Health Sciences University, Ponce, Puerto Rico, USA, psm.edu; ^5^ Department Head at Restorative Dentistry, Universidade Nove de Julho (UNINOVE), São Paulo, Brazil, uninove.br; ^6^ Department of Prosthodontics, ICT/SJC, UNESP, São José dos Campos, Brazil, unesp.br

**Keywords:** bond strength, milled, universal resin cement

## Abstract

**Objectives:**

This in vitro study evaluated the push‐out bond strength of two types of intraradicular fiber posts: conventional customized anatomical posts and milled fiber posts, cemented with two different strategies.

**Methods and Materials:**

Sixty bovine incisors with roots sectioned at the cementoenamel junction and standardized to a length of 15 mm were endodontically treated. All specimens were randomly assigned to four groups (*n* = 15) based on post type and cementation strategy; customized anatomical post cemented with self‐adhesive resin cement (RelyX U200, G1); customized anatomical post cemented with dual‐cure resin cement (RelyX Universal resin cement, G2); milled fiber post cemented with self‐adhesive resin cement (RelyX U200 resin cement, G3); and milled fiber post cemented with dual‐cure resin cement (RelyX Universal resin cement, G4). Push‐out bond strength measurements (MPa) were obtained from three root sections (cervical, middle, and apical) using a universal testing machine at a speed of 0.5 mm/min until failure. The interfaces and failure modes were analyzed using scanning electron microscopy (SEM). Statistical analysis included three‐way analysis of variance (ANOVA) and Tukey’s post hoc test to compare groups and root sections.

**Results:**

CAD/CAM‐milled posts (G3 and G4) exhibited significantly higher bond strength (mean PBS = 13.1 ± 3.9 MPa) compared to customized anatomical posts (G1 and G2) (mean PBS = 9.7 ± 2.8 MPa) (*p*  < 0.01). Bond strength varied significantly across root thirds (*p* = 0.034), with the apical and middle thirds demonstrating higher values than the cervical third. No significant difference in bond strength was observed between the two cementation strategies (*p* = 0.27).

**Conclusion:**

CAD/CAM‐milled fiber posts demonstrated higher push‐out bond strength than customized anatomical posts, regardless of the cementation strategy. However, these groups also exhibited a greater frequency of catastrophic failures, indicating that higher bond strength should not be interpreted as overall clinical superiority. Additional studies are needed to determine the clinical significance of these findings.

## 1. Introduction

The use of intraradicular posts is recommended for the rehabilitation of endodontically treated teeth with extensive coronal structure loss, particularly when less than 50% of the remaining coronal structure is present [1]. Traditionally, metal posts have been the primary clinical option in such cases; however, their inherent rigidity is associated with a high risk of root fracture and poor esthetic outcomes, especially when used with all‐ceramic restorations [2–4].

To overcome these limitations, customized anatomical fiber posts have emerged as a viable alternative. These posts, created by adapting a light‐curing resin composite around a prefabricated fiber post to conform to the internal root canal anatomy, exhibit a modulus of elasticity similar to that of dentin [5, 6]. This similarity promotes more favorable stress distribution and has been associated with a reduced incidence of catastrophic failures [7, 8]. Nevertheless, this technique often results in a thick, nonuniform cement layer that may incorporate voids and defects, contributing to debonding, one of the primary modes of failure in post‐retained restorations [9]. To reduce the thickness of the cement layer and improve adaptation, postanatomization has been proposed [10, 11].

In response, advances in CAD/CAM technology have enabled the fabrication of milled fiber posts that can be individually designed and produced as a single unit. These digitally fabricated posts eliminate the need for resin‐based customization, offering more precise adaptation to the canal walls, a more uniform cement layer, and potentially improved mechanical properties [12–14]. Despite these advantages, post‐retention remains dependent not only on design and adaptation but also on the properties and application of the luting agent used for cementation [15, 16]. Despite these advantages, CAD/CAM‐milled fiber posts are not without limitations. Their intimate adaptation to the root canal and reduced cement thickness may increase the stress transfer to the surrounding dentin during functional loading. Consequently, higher bond strength may not necessarily correspond to more favorable failure patterns, as stronger adhesive interfaces can redirect stresses toward the root structure, potentially resulting in catastrophic fractures. Therefore, evaluation of both bond strength and failure mode is essential when assessing the overall mechanical behavior of different post systems.

Post‐retention is influenced not only by post design and adaptation but also by the properties and application protocols of the luting system [15]. Recent evidence has demonstrated that different adhesive strategies and resin cements can significantly affect the push‐out bond strength of fiber‐reinforced posts to root dentin, particularly under intraradicular conditions [15]. In this context, the curing mechanism of resin cements plays a critical role. Dual‐cure resin cements are commonly recommended for post cementation because they combine chemical and light‐activated polymerization, ensuring more reliable curing in deeper regions of the canal where light transmission is limited [17]. Self‐adhesive resin cements simplify the clinical protocol by eliminating separate etching and bonding steps; however, their bonding effectiveness may depend on the interaction with universal adhesives and curing mode [17]. Additionally, comparative in vitro studies evaluating conventional, universal, and self‐adhesive resin cements for cementation of prefabricated and CAD/CAM‐milled fiber post‐and‐core systems have reported that cement type alone may not significantly influence bond strength when adequate postadaptation and standardized cementation protocols are employed [18, 19].

A previous study investigated the push‐out bond strength of root dentin using standard, relined, and CAD/CAM glass fiber post‐and‐core systems luted with two resin composite adhesive cements, one conventional and one universal, as well as a self‐adhesive resin cement. The authors found no significant differences in the bond strength among the cement types. However, that study did not directly compare CAD/CAM‐milled posts with customized anatomical posts in terms of intraradicular adaptation or bond strength across the root thirds, particularly when using a universal resin cement [18, 19]. To date, limited data are available comparing their performance with that of self‐adhesive cements, particularly in anatomically complex areas such as the apical third of the root canal [20].

Therefore, the present in vitro study aimed to compare the push‐out bond strength of CAD/CAM‐milled fiber posts and customized anatomical posts, each cemented with either a self‐adhesive (RelyX U200) or a universal (RelyX Universal) dual‐cure resin cement across three root thirds (cervical, middle, and apical) to assess the influence of canal region on cementation efficacy. The null hypotheses tested were (1) there is no significant difference in push‐out bond strength between CAD/CAM‐milled and customized anatomical posts, (2) there is no significant difference in push‐out bond strength between the two resin cement types, and (3) there is no significant difference in push‐out bond strength across the root thirds (cervical, middle, and apical).

## 2. Methods and Materials

Bovine incisors were selected because they provide standardized root dimensions, a relatively homogeneous dentin substrate, and consistent specimen availability, thereby reducing biological variability among experimental groups. Previous studies have demonstrated that bovine dentin is a suitable substitute for human dentin in laboratory bond strength investigations; however, differences in dentinal microstructure and tubule density should be considered when extrapolating these findings to clinical situations.

The sample size was determined using power analysis based on data from a previous study [12] that evaluated bond strength between different post systems and resin cements. A minimum of 15 specimens per group (total *N* = 60) was calculated to detect a medium effect size (*f* = 0.25) with a power of 80% and an alpha level of 0.05 using a three‐way analysis of variance (ANOVA) design. This ensured sufficient statistical power to detect differences among post types, cementation strategies, and root thirds. All residual periodontal tissues were meticulously removed using periodontal curettes, and the teeth were stored in a saline solution to maintain hydration until the experiment began. Crowns were sectioned horizontally with a carborundum disc (3M Oral Care, St. Paul, MN, USA) using a straight handpiece under constant irrigation, standardizing the root length to 15 mm, measured from the apex to the cementoenamel junction or as close as possible to the cervical region [12].

Root canals were instrumented manually using K‐files (sizes #45–#80, Maillefer, Ballaigues, Switzerland) and prepared using the step‐back technique with constant irrigation using 2.5% sodium hypochlorite (NaOCl, CanalPro, Coltene/Whaledent, Altstätten, Switzerland) [17]. After instrumentation, canals were cleaned with water‐soluble gel, dried with absorbent paper points, and obturated by a calibrated operator using the hybrid Tagger technique, which combines cold lateral condensation with thermo‐mechanical compaction to enhance gutta‐percha adaptation along the canal walls [21]. The canals were sealed with eugenol‐free temporary cement and stored for 7 days at 37°C in a saline solution (Becker Products Drug Hospitals, Campos Novos, SC, Brazil). Following storage, gutta‐percha was removed using Peeso reamers (sizes 2 and 3), and the post spaces were prepared with size 3 drills to a depth of 10 mm, maintaining a 5 mm apical seal and standardizing post space length [17]. Debris was removed by rinsing with 2 mL of 2.5% NaOCl, and the canals were dried with absorbent paper points.

### 2.1. Customized Anatomical Posts (Groups G1 and G2)

For Groups G1 and G2, prefabricated glass fiber posts (size #3) (3M Oral Care, St. Paul, MN, USA) were used. These posts were initially in their stock form and had not yet been customized to anatomical posts. They were first cleaned with 70% alcohol and air‐dried for 20 s prior to the adhesive application. Scotchbond Universal Adhesive (3M Oral Care, St. Paul, MN, USA) was applied with a microbrush for 20 s with Single Bond Universal adhesive for G1 and Scotchbond Universal Plus adhesive for G2 according to the manufacturer’s instructions.

To facilitate the fabrication of customized anatomical posts, the root canal walls were coated with a water‐soluble gel (KY Jelly, Johnson & Johnson, New Brunswick, NJ, USA), serving as a separating medium. This allowed for the intraoral shaping of the posts with the resin composite and their removal for complete light curing outside the canal. The gel was subsequently removed with 2.5% NaOCl, and the canals were dried using absorbent paper points [21].

Customized anatomical posts were performed by incrementally applying nanoparticulate composite resin Filtek Z350 XT–A2 (3M Oral Care, St. Paul, MN, USA) around the fiber posts. Light polymerization was performed intraorally for 20 s with the post inside the root canal using a LED curing unit Bluephase PowerCure with 1000 mW/cm^2^ (Ivoclar, Schaan, Liechtenstein), followed by an additional 40 s of curing outside the canal to ensure complete polymerization [14].

### 2.2. CAD/CAM‐Milled Posts (Groups G3 and G4)

For Groups G3 and G4, root canals were scanned internally using a Trios 4 intraoral scanner (3Shape, Copenhagen, Capital Region, Denmark). Scans were processed with Exocad software (Exocad GmbH, Darmstadt, Hesse, Germany), and the posts were fabricated using a 5‐axis milling unit (Ceramill Motion 2, Amann Girrbach, Austria). Posts were milled from Zantex blocks, a high‐performance polymer matrix reinforced with a dense three‐dimensional network of glass fibers. After milling, the posts were sandblasted with 100 µm aluminum oxide particles, cleaned with steam, and further disinfected with 70% ethyl alcohol [12].

### 2.3. Post‐Cementation Section

All posts were cemented according to the manufacturers’ instructions [22]. For groups receiving universal adhesive systems (G2 and G4), the adhesive was actively applied to the root canal walls using a microbrush for 20 s with continuous agitation to enhance monomer penetration. Excess solvent was removed by gentle air‐thinning for ~5 s using oil‐free compressed air directed from the canal orifice to avoid pooling within the canal. No light activation of the adhesive was performed prior to cementation in accordance with the manufacturer’s recommendations for use with dual‐cure resin cements.

Groups G1 and G3 were cemented using the self‐adhesive resin cement RelyX U200 according to the manufacturer’s recommendations. Groups G2 and G4 were cemented using RelyX Universal dual‐cure resin cement in combination with Scotchbond Universal Plus Adhesive. The cementation procedures were standardized for all groups with respect to adhesive application, seating pressure, excess cement removal, and polymerization protocol to minimize operator‐dependent variability. Light curing was then performed for 20 s from the coronal aspect using a Bluephase PowerCure LED unit (Ivoclar, Schaan, Liechtenstein). Chemical curing ensured complete polymerization in deeper regions of the canal, with complete polymerization achieved within 10 min, consistent with the dual‐cure mechanism. All specimens were subsequently stored in distilled water at 37°C for 7 days to simulate intraoral conditions.

### 2.4. Bond Strength Measurement Section

Each specimen was embedded in self‐cure acrylic resin blocks Vipiflash (VIPI, Pirassununga, São Paulo, Brazil) according to ISO 20795‐1 and sectioned perpendicularly to the long axis using a dental precision cutting machine Isomet 1000 (Buehler, Lake Bluff, Illinois, USA) under constant irrigation. Root thirds were obtained as 2 mm thick slices by sectioning the roots at defined locations to represent the cervical, middle, and apical regions. Sectioning was performed perpendicular to the long axis of the root to produce three distinct specimens per tooth: the cervical third, located at the coronal end of the 10 mm post space; the middle third, positioned midway along the post space; and the apical third, corresponding to the area near the apical extent of the post preparation. Push‐out bond strength testing was conducted using a universal testing machine (Kratos Dynamometers, Embu, São Paulo, Brazil) at a crosshead speed of 0.5 mm/min with a maximum compressive load of 700 N. A 0.9 mm diameter piston was centrally positioned on the post until displacement or failure occurred. The maximum failure load was recorded in Newtons (N) and converted to megapascals (MPa) by dividing the failure load by the bonded area (mm^2^) for each root third. The bonded area was calculated using the following formula:

A=πR1+R2R1−R22+h2A=πR1+R2R1−R22+h2,

where *A* = cemented area, π = 3.14, *R*_1_ = largest radius of the post (cervical region), *R*_2_ = smallest radius of the post (apical region), and *h* = thickness of the root section, standardized at 2 mm.

A 0.9 mm diameter stainless‐steel piston was selected because it provided contact with the post surface while minimizing contact with the surrounding dentin, thereby reducing the likelihood of introducing frictional forces during the push‐out test. This piston size has been widely adopted in previous push‐out bond strength investigations evaluating fiber posts.

For scanning electron microscopy (SEM) analysis, specimens were coated with a gold‐palladium layer (~15 nm thickness) using a sputter coater (MED‐020, Bal‐Tec). The specimens were examined using a scanning electron microscope (JEOL JSM‐6510, JEOL Ltd., USA), and photomicrographs were captured at 50× and 200× magnifications.

For statistical analysis, the Shapiro–Wilk test was used to assess data normality. Sample size calculation was performed using 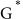
Power software (version X.X; Heinrich Heine University Düsseldorf, Düsseldorf, Germany). Each tooth was considered the experimental unit. Although three slices (cervical, middle, and apical thirds) were obtained from each tooth for push‐out testing, these slices were not treated as independent observations. Bond strength values were analyzed according to the root third within the same tooth to avoid pseudo‐replication. A three‐way ANOVA was performed to evaluate the effects of post type, cement type, and root third. When significant differences were found, Tukey’s post hoc test was applied to compare mean bond strength values between groups and root regions.

## 3. Results

This in vitro study evaluated the bond strength (PBS) in megapascals (MPa), considering the factors of post type, cementation strategy, and root thirds. The Shapiro–Wilk normality test confirmed that the PBS values followed a normal distribution (*p*  > 0.07). A three‐way ANOVA was performed to assess the effects of post type, cementation strategy, and root thirds on bond strength. A statistically significant interaction was observed between post type and root third (*p*  < 0.05), as shown in Table 1.

**Table 1:** Three‐factor analysis of variance (ANOVA) considering the factors post type, cementation strategy, and root thirds.

Factors				*p*
Post type				<0.001
Cementation strategy				0.279
Root thirds				0.034
Post type × cementation strategy				0.893
Post type × third				0.616
Cementation strategy × third				0.765
Post type × cementation strategy × third				0.713

The root thirds factor was statistically significant (*p* = 0.03), indicating a meaningful difference in PBS values between root levels. In contrast, the cementation strategy showed no statistically significant effect (*p* = 0.27), suggesting that the type of resin cement used did not influence bond strength.

Table 2 presents the results comparing post types. CAD/CAM‐milled fiber posts exhibited significantly higher mean bond strength than customized anatomical posts, with the difference being statistically significant (*p*  < 0.01). Both milled post groups (G3 and G4) outperformed the anatomical groups (G1 and G2), regardless of the resin cement used, confirming that post type played a greater role in adhesive performance than the cementation strategy. The milled group also showed a wider range of bond strength values, including both higher maximum and lower minimum values.

**Table 2:** Comparisons of bond strength between customized anatomical posts and milled fiber posts.

Descriptive statistic	Value	
	Anatomical	Milled
Validity	90	90
Mean ± SD	9.7 ± 2.8^A^	13.1 ± 3.9^B^
Minimum	2.8	0.9
Maximum	19.3	23.3
Tukey test		

*Note:* Reference values in MPa. Different letters indicate statistically significant differences for the post type factor.

Table 3 presents the Tukey post hoc results for the root third factor. The apical (12.1 ± 3.9 MPa) and middle thirds (11.7 ± 3.7 MPa) demonstrated statistically similar performance, while the cervical third (10.5 ± 3.7 MPa) exhibited significantly lower bond strength. These findings suggest that bond strength was influenced by the root location, with deeper regions (apical and middle) showing improved adhesive retention.

**Table 3:** Tukey test for the thirds factor.

Descriptive statistic	Value		
	Apical	Cervical	Middle
Validity	60	60	60
Mean ± SD	12.1 ± 3.9^A^	10.5 ± 3.7^B^	11.7 ± 3.7^A^
Minimum	4.3	2.8	0.9
Maximum	23.3	20.7	19.9

*Note:* Reference values in MPa. Different letters indicate statistical difference for the root thirds.

### 3.1. SEM

**Figure 1:**
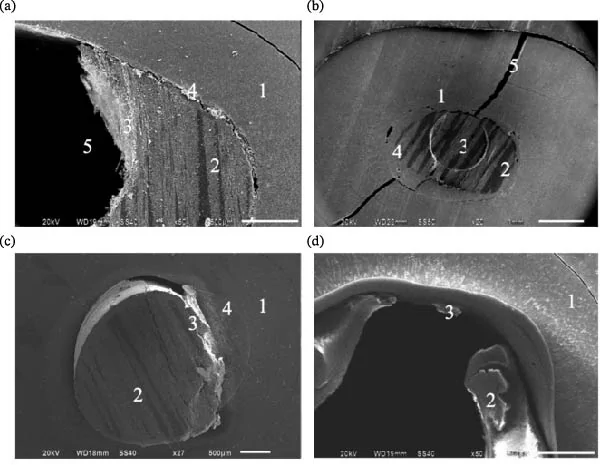
(a) Adaptation of the milled fiber post to the root dentin and cementation layer: (1) dentin, (2) Zantex, (3) fiber displacement, (4) cementation layer, and (5) failure area. (b) Catastrophic fracture of dentin: (1) dentin, (2) Zantex, (3) load application area, (4) cementation layer, and (5) fracture in dentin. (c) Cohesive failure in dentin showing fiber displacement in the post: (1) dentin, (2) Zantex, (3) fiber displacement, and (4) cementation layer. (d) Displacement of the post at the resin/cement interface: (1) dentin, (2) composite resin, and (3) remaining cement.

To evaluate the bonding interface and failure mode, microstructural analysis was conducted using SEM. Predominantly reparable adhesive failures at the resin cement line were observed in Groups G1 and G2, as shown in Figure 1a. These groups exhibited thin cement layers with good adaptation between the post and canal wall, suggesting effective frictional retention.

In contrast, catastrophic failures were more frequent in Groups G3 and G4, often involving fractures of the remaining tooth structure or the post itself, as depicted in Figure 1b. These groups withstood greater loading forces prior to failure, and dentin fractures were observed while the adhesive interface remained intact.

In the milled post groups, cohesive failures within dentin were predominant (Figure 1c). Conversely, in the prefabricated and customized anatomical post groups, a higher percentage of failures occurred at the interface between the post and the resin cement (Figure 1d).

## 4. Discussion

The findings of this study indicate that root location significantly affects the adhesive interface, likely due to variations in light‐curing efficiency, dentin structure, and access for cementation. The significant effect of the root segment on bond strength corroborates earlier findings related to the behavior of milled posts [22]. Typically, the cervical third demonstrates higher bond strength due to improved light penetration during polymerization, better accessibility, and superior dentin quality. In contrast, the apical third often presents clinical limitations, including restricted access, reduced polymerization efficacy, and anatomical variations in dentin [22, 23]. Interestingly, the present findings differ from several previous investigations that reported higher bond strength values in the cervical region. Differences in specimen preparation, post systems, resin cements, testing methodologies, and root canal standardization may partially explain these discrepancies. Because these variables differ considerably among studies, a direct comparison should be interpreted cautiously. Additional standardized investigations are necessary to clarify whether regional bond strength differences are primarily related to postadaptation, cement properties, dentin characteristics, or experimental methodology. However, in the present study, the apical third exhibited the highest mean bond strength, followed closely by the middle third, while the cervical third showed the lowest values. Statistically, there was no significant difference between the apical and middle thirds. Although the cervical third demonstrated significantly lower bond strength than the middle and apical thirds, the present study did not directly evaluate variables such as cement thickness, internal adaptation, void distribution, dentin morphology, or mechanical interlocking. Consequently, the mechanisms responsible for these regional differences cannot be established from the present data. The explanations discussed herein should therefore be interpreted as hypotheses supported by previous investigations rather than direct experimental findings.

Although SEM analysis provided valuable qualitative information regarding failure patterns, it was not designed to compare the failure modes among individual root thirds. Consequently, the SEM findings cannot explain the lower bond strength observed in the cervical region and should be interpreted independently from the push‐out bond strength results.

The performance of CAD/CAM‐milled fiber posts, as demonstrated by the higher mean bond strength values observed in Groups G3 and G4, is consistent with previous findings on apical root thirds [22]. A systematic review and meta‐analysis reported that CAD/CAM‐milled fiber posts provide improved fracture and bond strength compared to those of customized anatomical posts [24]. This improvement has been attributed to their enhanced adaptation and reduced cement layer thickness, contributing to a more favorable mechanical performance [14]. However, in the present study, although milled posts showed higher mean bond strength, they were also associated with a greater frequency of catastrophic failures, which warrants a cautious interpretation of the term “superior.” The precise fit of milled posts likely facilitates more consistent integration with root dentin and may reduce stress concentrations through a more uniform cement interface, which is critical for long‐term retention, but these benefits must be weighed against the potential risk of structural compromise under load. It should be emphasized that internal adaptation, cement thickness, and gap formation were not quantitatively evaluated in the present study. Therefore, any relationship between bond strength and adaptation should be interpreted as a possible explanation supported by previous investigations rather than as a direct finding of this study. Future investigations incorporating microcomputed tomography, optical microscopy, or image analysis techniques are needed to establish correlations between postadaptation, cement thickness, and adhesive performance.

In contrast, the lower bond strength observed in Groups G1 and G2 (customized anatomical posts) may be attributed to the increased number of adhesive interfaces introduced during the anatomization process. These additional interfaces can be more susceptible to adhesive failure under mechanical loading. Previous research evaluating various customized anatomical post systems found that CAD/CAM‐milled fiberglass post systems demonstrated a superior BS and adaptation to the root dentin compared to the conventional fiberglass post [25].

Failure modes were evaluated globally rather than according to individual root thirds. Consequently, correlations between regional bond strength values and specific failure patterns could not be established. Future studies should investigate failure modes separately for cervical, middle, and apical regions to better understand the mechanical behavior of different post systems along the root canal.

Although statistically significant differences were identified among root thirds, the magnitude of these differences was relatively small. Therefore, their clinical significance remains uncertain, particularly considering that this investigation was performed under controlled laboratory conditions without thermal aging or functional cyclic loading. Clinical studies are necessary to determine whether these differences translate into meaningful long‐term restorative outcomes.

Interestingly, in the present study, the cementation strategy did not significantly affect bond strength. This suggests that both RelyX U200 and RelyX Universal resin cements can achieve comparable adhesive performance when the proper technique is followed. RelyX U200 was used in combination with Single Bond Universal Adhesive (lacking MDP), while RelyX Universal was used with Scotchbond Universal Adhesive (containing MDP: 10‐Methacryloyloxydecyl dihydrogen phosphate). MDP is known to enhance chemical adhesion by forming strong bonds with hydroxyapatite in the tooth structure, potentially influencing cement performance. Although some studies have reported advantages of RelyX U200 in terms of versatility and user‐friendliness, the present results suggest that post type and adaptation play a more critical role in achieving optimal bond strength than the specific cement or the presence of MDP [19, 22]. These findings suggest that, when standardized cementation protocols and adequate postadaptation are achieved, cement chemistry alone may play a secondary role in bond performance compared with factors such as post design and interfacial fit [25].

In the present study, higher mean push‐out bond strength values were revealed in the apical and middle thirds compared with the cervical third, which contrasts with the widely reported trend of greater bond strength in coronal regions due to improved light penetration and access during cementation. Traditionally, enhanced bond strength in the cervical third has been attributed to more favorable dentin tubule orientation, better polymerization of resin in areas with direct light exposure, and easier access for adhesive application. However, several factors in the present study may have contributed to an inverse or uniform pattern of retention. First, the uniform adaptation of both milled and customized posts in the deeper regions may have resulted in more consistent mechanical interlocking within the middle and apical thirds, particularly if the designed post geometry and canal preparation reduced microgaps and improved contact with dentin walls. Second, cement layer thickness can vary by region; a thinner and more uniform cement layer may occur in deeper regions when posts closely match the canal anatomy, reducing voids and stress concentrators that can weaken the interface. Third, the experimental conditions used standardized post spaces and controlled irrigation, which may have minimized variables such as the smear layer or residual moisture that disproportionately affect the cervical region.

It is important to note that this study was conducted under controlled in vitro conditions, and the findings do not imply direct clinical applicability. Factors such as thermal cycling, occlusal loading, and long‐term degradation were not addressed and warrant further exploration. Future research should include in vivo studies to evaluate the clinical performance of milled versus customized anatomical posts as well as the impact of different polymerization protocols and resin cement chemistries on bonding outcomes.

Although previous studies have evaluated the bond strength of customized anatomical fiber posts and CAD/CAM‐fabricated fiber posts individually, direct comparisons between these restorative approaches using different cementation strategies remain limited. Furthermore, the influence of the root region on push‐out bond strength and failure patterns has not been fully clarified. Therefore, this study was designed to compare the push‐out bond strength and failure modes of anatomically customized and CAD/CAM‐milled fiber posts cemented using two contemporary adhesive strategies.

## 5. Conclusion

Within the limitations of this in vitro study, CAD/CAM‐milled fiber posts demonstrated significantly higher push‐out bond strength than customized anatomical posts, regardless of the cementation strategy employed. Bond strength differed among root thirds, with lower values observed in the cervical region. However, CAD/CAM‐milled posts also exhibited a greater frequency of catastrophic failures, indicating that increased bond strength should not be interpreted as overall clinical superiority. Because variables such as internal adaptation, cement thickness, and gap formation were not directly measured, no causal relationship can be established between these factors and bond strength. Further laboratory and clinical investigations are required to confirm these findings.

## Author Contributions

Camila Orlinda Andrade Gusmão contributed to conception, design, data acquisition, drafted the manuscript, and critically revised the manuscript. Vanessa Inácio Celestrino, Alfredo Mikail Melo Mesquita, Carlos Alberto Jurado, Rubens Nisie Tango, and Washignton Steagall contributed to conception, data acquisition, drafted the manuscript, and critically revised the manuscript. Franciele Floriani contributed to the design, drafted the manuscript, critically revised the manuscript, and contributed to data interpretation. Amirali Zandinejad contributed to conception, drafted the manuscript, critically revised the manuscript, and contributed to data interpretation.

## Funding

The authors received no specific financial support for the research, authorship, and/or publication of this article.

## Disclosure

All authors gave their final approval and agreed to be accountable for all aspects of the work.

## Conflicts of Interest

The authors declare no conflicts of interest.

## Data Availability

The raw data supporting the findings of this study are currently available to the authors.
